# High-concentration peat drives divergent transcriptomic responses to enhance saline-alkaline tolerance and phytoremediation in two *Suaeda* species

**DOI:** 10.3389/fpls.2026.1761230

**Published:** 2026-02-27

**Authors:** Li Zhou, Zhaokui Du, Pengpeng Lv, Zitong Wang, Chaonan Cai, Junmin Li

**Affiliations:** 1 Zhejiang Key Laboratory for Restoration of Damaged Coastal Ecosystems, School of Life Sciences, Taizhou University, Zhejiang, Taizhou, China; 2 Zhejiang Provincial Key Laboratory of Plant Evolutionary Ecology and Conservation, School of Life Sciences, Taizhou University, Taizhou, China; 3 School of Advanced Study, Taizhou University, Taizhou, China

**Keywords:** halophytes, peat, phytoremediation, salt-responsive genes, *Suaeda glauca*, *Suaeda salsa*

## Abstract

**Introduction:**

Soil salinization threatens global land use and food security, and halophytes combined with peat amendments are promising for saline-alkali soil remediation.

**Methods:**

Here, we integrated transcriptomic and physiological analyses to investigate the adaptive responses of *Suaeda glauca* and *S. salsa* grown in saline-alkaline soils amended with peat at 0, 6, or 18 g/kg.

**Results and discussion:**

Our results showed that a high peat concentration (18 g/kg) significantly improved salt tolerance and biomass accumulation in both species through distinct species-specific strategies. *S. glauca* upregulated growth-related pathways (e.g., nitrogen metabolism, and tricarboxylic acid cycle) mediated by bHLH and bZIP transcription factors (TFs), whereas S. salsa activated stress-mitigating secondary metabolism (e.g., flavonoids, phenylpropanoids, anthocyanins) regulated by MYB and NAC TFs. A conserved response across both species was the downregulation of genes involved in amino acid degradation, which helps conserve nitrogen for osmoprotection. RT-qPCR analysis confirmed the reliability of the RNA-seq data. This study identified 18 g/kg as the optimal peat concentration, uncovers species-specific adaptive mechanisms in halophytes, and lays a foundation for the precisely selection of halophyte-peat combinations in saline-alkaline soil remediation.

## 1 Introduction

According to the Food and Agriculture Organization of the United Nations, approximately 424 million hectares of topsoil (0–30 cm) and 833 million hectares of subsoil (30–100 cm) are affected by salinity worldwide (FAO, 2021). Soil salinization is a major constraint on land use efficiency and poses a serious threat to global food security, emerging as one of the most pressing environmental and socio-economic challenges (Hassani et al., 2020; Xu et al., 2023). Therefore, understanding the specific mechanisms of phytoremediation in saline soils and developing effective remediation strategies are both urgent and essential.

Halophytes, which can survive and reproduce in environments with salt concentrations exceeding 200 mM NaCl, constitute about 1% of the world’s flora (Flowers and Colmer, 2008). These species are well-adapted to saline-alkaline conditions and are divided into three categories: euhalophytes, salt-exclusion halophytes, and recretohalophytes (Grigore and Vicente, 2023). *Suaeda glauca* and *S. salsa* are typical succulent euhalophytes with a wide geographical distribution. In China, for instance, the two species are commonly found in the northeast, northern, and northwest regions, including provinces such as Shandong, Jiangsu, and Zhejiang (Feng et al., 2023b). *S. glauca* is recognized as a pioneer species in saline-alkali lands, capable of tolerating salinity up to 600 mM NaCl. *S. salsa* is considered an indicator species for saline–alkaline environments and can tolerate at least 400 mM NaCl (Cui et al., 2024; Li et al., 2023; Wang et al., 2021). Numerous studies have demonstrated the effectiveness of halophytes in revegetating and remediating of salt-affected soils (Flowers et al., 2010; Li et al., 2025; Shabala, 2013). For example, *S. salsa* was reported to reduce soil Na^+^ content by 4.5% at a depth of 20–30 cm when grown at a density of 15 plants per square meter within a single growing season (Zhao, 1991). Moreover, *S. salsa* increased soil organic matter and total nitrogen levels by 43% and 18%, respectively (Lin et al., 2005). Comparative genomic and transcriptomic studies have shown that *S. salsa* and *S. glauca* use different adaptive strategies under saline-alkali stress, involving the coordinated regulation of multiple metabolic pathways (Song et al., 2022; Yan et al., 2024).

In recent years, soil amendments such as biochar, crop residue, and peat have been increasingly applied to improve saline soil conditions (Yang et al., 2021; Zhao et al., 2024). Peat, a spongy material formed from the partial decomposition of plant-based organic matter, can substantially improve soil organic matter in degraded soils (Fu et al., 2021). For example, peat application has been shown to reduce soil salt concentrations in the root zone of coastal saline soils (Wang et al., 2014). Additionally, Yang et al. (2021) found that burying a peat layer in the saline soil is an effective method to reduce infiltration in the upper soil layers and simultaneously inhibit phreatic evaporation (Yang et al., 2021). Despite the proven efficacy of peat amendments and halophyte phytoremediation, existing studies primarily focus on single peat concentrations or individual halophyte species (Yang et al., 2021; Fu et al., 2021), with few investigating how divergent halophytes respond to gradient peat additions. Previous comparative studies on *S. glauca* and *S. salsa* have only addressed their intrinsic saline-alkali adaptation strategies (Song et al., 2022; Yan et al., 2024); however, the species-specific responses to varying peat concentrations, especially the molecular mechanisms underlying plant-peat interactions, remain unclear. This knowledge gap hinders the precise selection of halophyte-peat combinations for tailored saline-alkaline soil remediation.

Here, we utilized a two-factor full factorial experimental design (two *Suaeda* species × three peat concentrations, 0, 6, and 18 g/kg) to systematically compare their transcriptomic responses. This design aimed to 1) determine the optimal peat concentration for the phytoremediation of saline-alkaline soils; 2) assess whether halophyte cultivation enhances the effectiveness of peat amendments; and 3) explore the differential responses between *S. salsa* and *S. glauca*, two halophytic species with distinct strategies for adapting to saline stress. Our findings not only identify critical salt-responsive genetic markers in halophytes but also provide a practical framework for improving coastal saline soil remediation.

## 2 Materials and methods

### 2.1 Plant materials and soil substrate

This study was conducted at the Jiaojiang Campus of Taizhou University, Zhejiang Province, China. Seeds of *S. glauca* and *S. salsa* were procured from the Qiaohua Tamarix Planting Co., Ltd., Shandong, China. In April 2024, the seeds of the two halophytes were surface-sterilized with a 0.5% NaClO solution for 15 minutes, washed three times with deionized water, and sown in prepared soil to germinate. They were kept in the greenhouse at a temperature of 25/20 °C with a relative humidity of 60%, under 16 h day/8 h night conditions.

In April 2024, saline-alkaline soil was collected from saline-alkali land in the Taizhou Economic Development Zone in Zhejiang, China (121°34′55″ E, 28°31′29″ N). This area is located on the south-eastern coast and has a typical subtropical monsoon climate. This has resulted in patterns of low-lying terrain and strong tidal action, as well as saline-alkali land, which have been influenced by evaporation and precipitation. The average annual temperature is 18 °C, and the annual rainfall is 1400 to 1800 mm. The soil was air-dried, sieved (2 mm mesh), and homogenized to remove coarse particles to facilitate plant growth.

The peat was supplied by Taizhou Agricultural Trade Co., Ltd. in Zhejiang, China. It was made from moss residues and had a pH value between 4 and 6. It contained over 95% organic matter and had a conductivity of 0.1 mS cm^−1^ (Fu et al., 2021).

### 2.2 Experiment design

To evaluate the effects of peat and halophytes in the phytoremediation of saline-alkaline soil, two halophytes (*S. glauca* and *S. salsa)* were cultivated under three different peat concentrations (0, 6, and 18 g/kg soil) following previous studies (Duan et al., 2023; Fu et al., 2021). The low concentration (6 g/kg) was derived by converting the optimal field application rate of wood peat for saline-sodic soil (19.57–23.95 t·ha^-1^, Duan et al., 2023) using a standard conversion coefficient (1 t·ha^-1^ ≈ 0.385 g/kg soil), yielding a converted pot concentration range of 7.53–9.21 g/kg. The high concentration (18 g/kg) was consistent with the pot-validated effective concentration (18.5 g/kg, Fu et al., 2021), which has been proven to significantly improve degraded soil properties and promote plant growth. Soil properties were identical to those reported in our previous study (Wang et al., 2025b). Uniformly grown seedlings were transplanted into plastic pots (10.5 cm×14 cm), each containing 1 kg of pretreated field soil, with one plant per pot. Plants were grown in a greenhouse under controlled conditions (28 °C light for 16 h, 18 °C dark for 8 h, and approximately 45% relative humidity). All pots were watered daily with the same amount of tap water.

### 2.3 Measurement of chlorophyll and growth parameters

Leaf chlorophyll content was determined using the 80% acetone method. The extracted solution was analyzed using a multifunctional enzyme marker (Readmax 1900, Flash, Shanghai, China) at wavelengths of 645 and 663 nm. The contents of chlorophyll a (C*_a_*) and chlorophyll b (C*_b_*) were calculated as follows: C*_a_* = 12.72×A663-2.59×A645, C*_b_* = 22.88×A645-4.67×A663 (Lichtenthaler and Wellburn, 1982). Three leaves per plant from four randomly selected plants were measured repeatedly.

### 2.4 Sample collection, RNA extraction, and transcriptome sequencing

Four fresh leaves per plant were sampled, and every three plants were pooled to form one biological replicate. Each treatment included three biological replicates. All samples were immediately frozen in liquid nitrogen and then stored at −80 °C until use.

Total RNA was extracted from 80 mg of leaf tissue using the ethanol precipitation protocol and the CTAB PBIOZOL reagent. RNA concentration was quantified using a NanoDrop 2000 spectrophotometer (Thermo Fisher Scientific, CA, USA), and RNA quality was assessed using the Bioanalyzer 2100 system (Agilent Technologies, CA, USA). Sequencing libraries were prepared using NEB Next^®^ Ultra^™^ RNA Library Prep Kit for Illumina^®^ (NEB, USA) following the manufacturer’s instructions. Subsequently, libraries were sequenced on an Illumina Nova Seq 6000 (Illumina Inc., San Diego, CA, USA) to produce 150 bp paired-end reads (Novogene, China).

### 2.5 Transcriptome data analysis

Initially, clean reads were obtained by removing reads containing adapters, poly-N, and low-quality reads from raw reads. At the same time, Q20, Q30, and GC content of the clean reads were calculated, ensuring high-quality clean reads for downstream analyses. Then, clean reads were used for transcriptome *de novo* assembly using Trinity (v2.6.6) with min_kmer_cov set to three and min_glue set to four, along with all the other parameters being set to default, producing reference sequences for the downstream analysis (Grabherr et al., 2011). The longest transcripts within each cluster were regarded as a unigene to eliminate redundant sequences. The function of these unigenes were annotated in the following databases: NCBI non-redundant protein sequences (Nr, e-value = 1e-5), NCBI non-redundant nucleotide sequences (Nt, e-value = 1e-5), Protein family (Pfam, e-value = 0.01), Clusters of Orthologous Groups of proteins (KOG/COG, e-value = 1e-5), A manually annotated and reviewed protein sequence database (Swiss-Prot, e-value = 1e-5), KEGG Ortholog database (KO, e-value = 1e-5), and Gene Ontology (GO, e-value = 1e-6) databases.

Differentially expressed genes (DEGs) between groups were identified using the DESeq 2 (1.26.0) (Love et al., 2014) with the thresholds of *p*<0.05 and |log_2_(FoldChange)| ≥ 1. GO and KEGG pathway enrichment analyses were performed using the cluster Profile R package v3.10.1 to infer the putative functions of the DEGs. GO terms and KEGG pathways with *p* < 0.05 were considered significantly enriched (Mao et al., 2005). TBtools was used to create gene expression heatmaps (Chen et al., 2020). Raw transcriptome sequencing data have been deposited in the NCBI Sequence Read Archive (SRA) under BioProject accession number PRJNA1189186.

### 2.6 Harvest and biomass measurement

After two months, the entire plants were harvested and washed with sterilized distilled water. Then, the plants were carefully separated into leaves, stems, and shoots. Each component was then dried at 70 °C until a constant weight was reached, after which the biomass was accurately measured using a precision balance.

### 2.7 Real-time quantitative PCR validation

The RNA samples used for RT-qPCR were consistent with those for RNA-seq. Total RNA was converted to complementary DNA (cDNA) using FastKing RT Kit (With gDNAase) (Tiangen, China) according to the manufacturer’s instructions. The qPCR was performed on a CFX Connect Real-time system (Bio-Rad, USA) via a FastFire qPCR premix (SYBR Green) kit (Tiangen, China) followed previously established protocols (Zhou et al., 2023). Three biological replicates and three technical replicates were analyzed with the *EF1* gene as an internal control. Relative expression was calculated using the 2−ΔΔCT method (Livak and Schmittgen, 2001). The primers for RT-qPCR are listed in **Supplementary Table 1**.

### 2.8 Statistical analysis

Statistical data analysis was performed using the Statistical Product and Service Solution (SPSS) software (v16.0). The effects of peat addition on chlorophylls and growth parameters were analyzed using one-way analysis of variance (ANOVA). Subsequently, Duncan’s multiple range test was performed at a 0.05 confidence level to examine differences among treatments.

## 3 Results

### 3.1 Effects of peat addition on the growth of two halophytes

**Figure 1:**
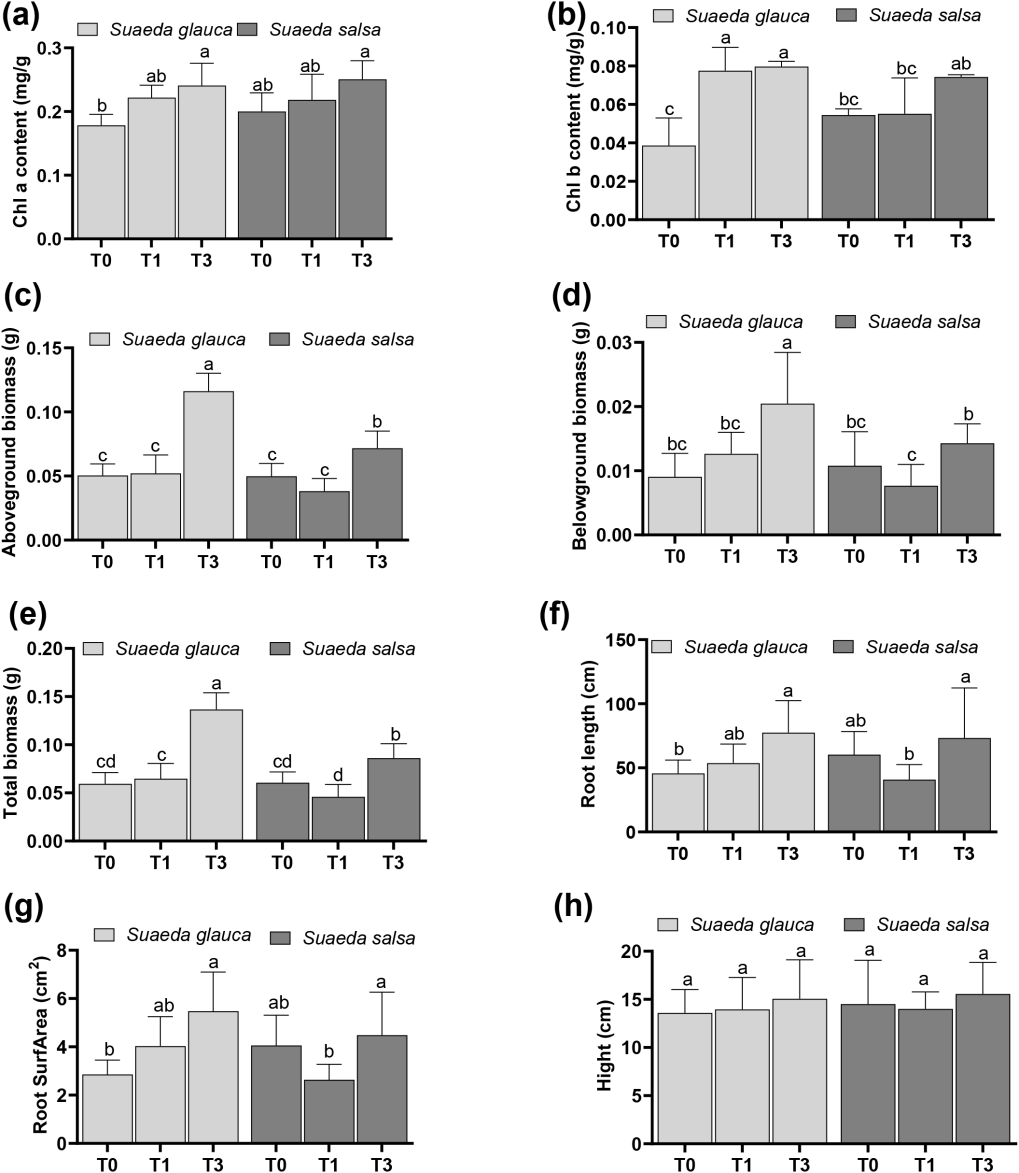
Effects of peat addition on the growth of halophytes. **(a)** chlorophyll a; **(b)** chlorophyll b; **(c)** aboveground biomass; **(d)** belowground biomass; **(e)** total biomass; **(f)** root length; **(g)** root surface area (SurfArea); **(h)** plant height. T0, T1, and T3 represent peat additions of 0, 6, and 18 g/kg of soil, respectively. Different lowercase letters indicate significant differences among treatments (*p* < 0.05, Duncan’s multiple range test).

Peat addition significantly increased chlorophyll a and b contents of *S. glauca*, while it had no significant effect on the chlorophyll a content in *S. salsa* (**Figures 1a, b**; **Table 1**). A significant interaction was observed between peat addition and plant species for chlorophyll b content (**Table 1**), indicating that the effect of peat on chlorophyll b accumulation varies with plant species. At the low peat concentration (6 g/kg), no significant effects were detected on the growth of either *S. glauca* or *S. salsa*. In contrast, the high peat concentration (18 g/kg) significantly increased aboveground, belowground, and total biomass in both species (**Figures 1c–e**). *S. glauca* exhibited significantly higher aboveground, belowground, and total biomass than *S. salsa*. High peat concentration also significantly increased root length and root surface area in *S. glauca* (**Figures 1f, g**), although no significant effect was observed on plant height (**Figure 1h**). Furthermore, a significant interaction between peat addition and plant species was found for both aboveground and total biomass (**Figures 1c, e**; **Table 1**).

**Table 1:** Results of linear models testing the effects of peat addition on the growth parameters of two species.

Growth parameter	Source of variation	d.f.	F	P
Total biomass	Peat (control vs low vs high)	2	50.08	0
	Species (*S.glauca* vs *S. salsa*)	1	21.05	0
	Peat × Species	2	8.69	**0.001**
Aboveground biomass	Peat (control vs low vs high)	2	51.66	0
	Species (*S.glauca* vs *S. salsa*)	1	21.57	0
	Peat × Species	2	8.90	**0.001**
Belowground biomass	Peat (control vs low vs high)	2	7.55	**0.002**
	Species (*S.glauca* vs *S. salsa*)	1	2.79	0.106
	Peat × Species	2	2.36	0.113
Plant height	Peat (control vs low vs high)	2	0.53	0.593
	Species (*S.glauca* vs *S. salsa*)	1	0.17	0.679
	Peat × Species	2	0.05	0.951
Root length	Peat (control vs low vs high)	2	0.01	**0.012**
	Species (*S.glauca* vs *S. salsa*)	1	1.26	0.918
	Peat × Species	2	2.40	0.301
Root surface area	Peat (control vs low vs high)	2	5.74	**0.008**
	Species (*S.glauca* vs *S. salsa*)	1	0.84	0.368
	Peat × Species	2	3.52	**0.043**

Bold P values indicate statistically significant effects (P< 0.05).

### 3.2 Overview of transcriptome data

A total of 116.77 Gb clean reads were generated, with an average of 6.5 Gb per library, a mean Q30 percentage of 94.39%, and a mean GC percentage of 43.09% (**Supplementary Table 2**). As genomes of *S. glauca* and *S. salsa* were not available, the clean reads were assembled using Trinity, resulting in 81,510 unigenes for subsequent analysis. The assembled unigenes ranged in length from 301 to 15,968 bp, with an average length of 1,154 bp, and an N50 value of 1,777 bp (**Supplementary Figure 1a**). All unigenes were aligned against seven databases based on sequence similarity using BLASTX. As a result, 49.48%, 41.3%, 40.68%, 40.68%, 39.58%, 20.91%, and 17.23% of the unigenes were annotated in the NR, Swiss-Prot, GO, PFAM, NT, KO, and KOG databases, respectively. In total, 55,197 (67.71%) unigenes were annotated in at least one of the databases, and 4,219 (5.17%) unigenes were annotated across all seven databases (**Supplementary Figure 1b**).

### 3.3 DEGs of two halophytes under different peat concentrations

**Figure 2:**
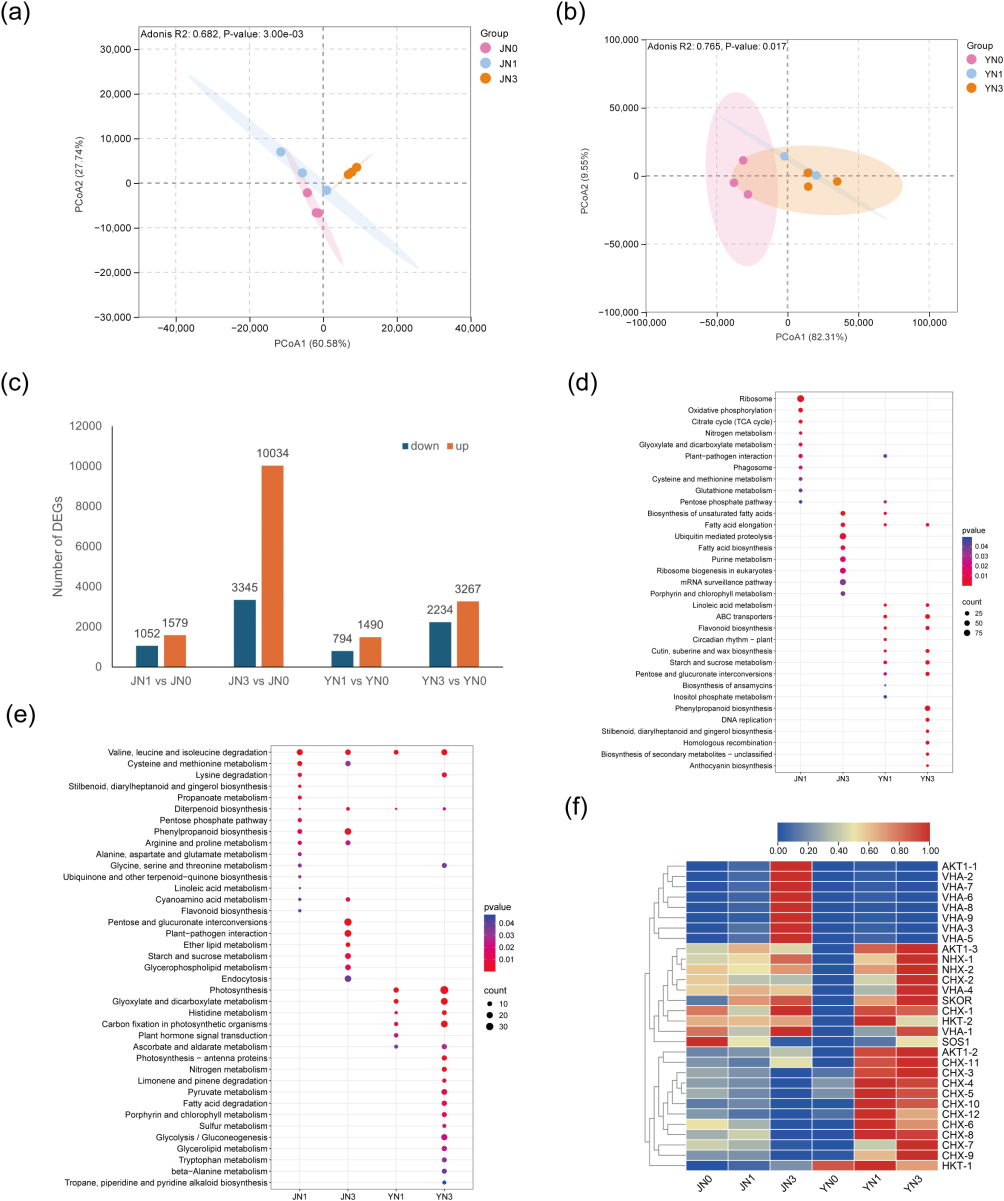
Transcriptome analysis of two halophytes in response to peat addition during saline-alkaline soil remediation. **(a, b)** PCoA plots for *S. glauca* and *S. salsa*, respectively; **(c)** DEGs; **(d, e)** KEGG enrichment analysis of upregulated and downregulated DEGs, respectively. **(f)** heatmap of the expression of Na+/K+ transporter genes. JN0, JN1, and JN3 represent *S. glauca* under 0, 6, and 18 mg/kg peat addition, respectively. YN0, YN1, and YN3 represent *S. salsa* under 0, 6, and 18 mg/kg peat addition, respectively.

Principal component analysis (PCA) and principal coordinates analysis (PCoA) revealed that peat addition caused significant changes in gene expression profiles in both *S. glauca* (**Figure 2a**; **Supplementary Figures 2a, c**) and *S. salsa* (**Figure 2b**; **Supplementary Figures 2b, d**), with a significant difference between low and high peat treatments (*p* < 0.05). DEGs were identified based on the criteria of |log_2_(FoldChange)| ≥ 1 and *p* < 0.05. In *S. glauca*, 2,631 DEGs (1,579 upregulated and 1,052 downregulated) and 13,379 DEGs (10,034 upregulated and 3,345 downregulated) were identified under low and high peat concentrations, respectively. In *S. salsa*, 2,284 DEGs (1,490 upregulated and 794 downregulated) and 5,501 DEGs (3,267 upregulated and 2,234 downregulated) were identified under low and high peat concentrations, respectively (**Figure 2c**). In both species, the number of DEGs increased with the increasing levels of peat concentrations, with more genes being upregulated and fewer downregulated. High peat levels caused more DEGs than low levels. Notably, only 73 DEGs (0.4%) were between the two species across both low and high levels of peat addition (**Supplementary Figure 2c**). In summary, peat addition has exerted significant and concentration-dependent regulatory effects on the gene expression of *S. glauca* and *S. salsa*, and the transcriptomic responses of the two species to this treatment are highly species-specific. This difference may reflect their distinct molecular strategies for adapting to peat-amended environments.

Additionally, KEGG and GO enrichment analyses of the DEGs showed distinct responses to peat addition between *S. glauca* and *S. salsa* (**Figure 2d**; **Supplementary Figure 3**). In *S. glauca*, upregulated DEGs were mainly enriched in growth-related pathways. At low peat concentrations, genes related to ribosome, tricarboxylic acid (TCA) cycle, and nitrogen metabolism pathways were activated. In contrast, high peat concentrations induced genes related to fatty acid biosynthesis, ribosome biogenesis in eukaryotes, and porphyrin and chlorophyll metabolism pathways. In *S. salsa*, the upregulated DEGs were mainly enriched in secondary metabolite-related pathways. Both low and high peat treatments activated genes involved in linoleic acid metabolism, flavonoid biosynthesis, and cutin, suberine, and wax biosynthesis. High peat concentrations further induced the biosynthesis of phenylpropanoids, anthocyanins, and other secondary metabolites (**Figure 2d**).

In *S. glauca*, the downregulated DEGs were mainly enriched in amino acid degradation and metabolism, as well as secondary metabolites-related pathways. Specifically, both low and high peat concentrations caused the expression of genes related to the degradation of valine, leucine, and isoleucine; the metabolism of cysteine, methionine, arginine, proline, and cyanoamino acid; as well as the biosynthesis of phenylpropanoids and diterpenoids. In *S. salsa*, the downregulated DEGs were mainly enriched in growth-related pathways. Both peat treatments caused the expression of genes involved in photosynthesis, carbon fixation in photosynthetic organisms, etc. (**Figure 2e**).

### 3.4 Salt-responsive Na^+^/K^+^ transporter genes exhibited species-specific expression patterns

A total of 30 cation transporter genes associated with Na^+^ homeostasis and K^+^ absorption were identified among DEGs, covering 7 functional families: 12 *CHX* (cation/H^+^ exchanger), 9 *VHA* (vacuolar H^+^-ATPase), 3 *AKT1* (inward rectifying K^+^ channel), 2 *NHX* (vacuolar Na^+^/H^+^ antiporter), 2 *HKT* (high-affinity K^+^ transporter), 1 *SOS1* (plasma membrane Na^+^/H^+^ antiporter), and 1 *SKOR* (stelar K^+^ outward rectifier). The expression patterns of these transporters showed pronounced species-specific responses to peat addition. In *S. glauca*, 5 *VHA* genes were significantly upregulated under low peat concentration, whereas no other transporter families exhibited differential expression. Under high peat concentration, 19 transporters were differentially expressed, including 10 downregulated genes (8 *CHX*, 1 *SOS1*) and 9 upregulated genes (7 *VHA*, 1 *SKOR*, 1 *AKT1*). In *S. salsa*, multiple transporter families were upregulated under the low peat treatment, including 5 *CHX*, 2 *AKT1*, 2 *NHX*, 2 *VHA*, and 1 *HKT*. Under high peat concentration, all 9 detected transporters were upregulated, covering *CHX* (3), *AKT1* (2), *NHX* (2), *VHA* (1), and *HKT* (1) (**Supplementary Figure 2d**).

**Figure 3:**
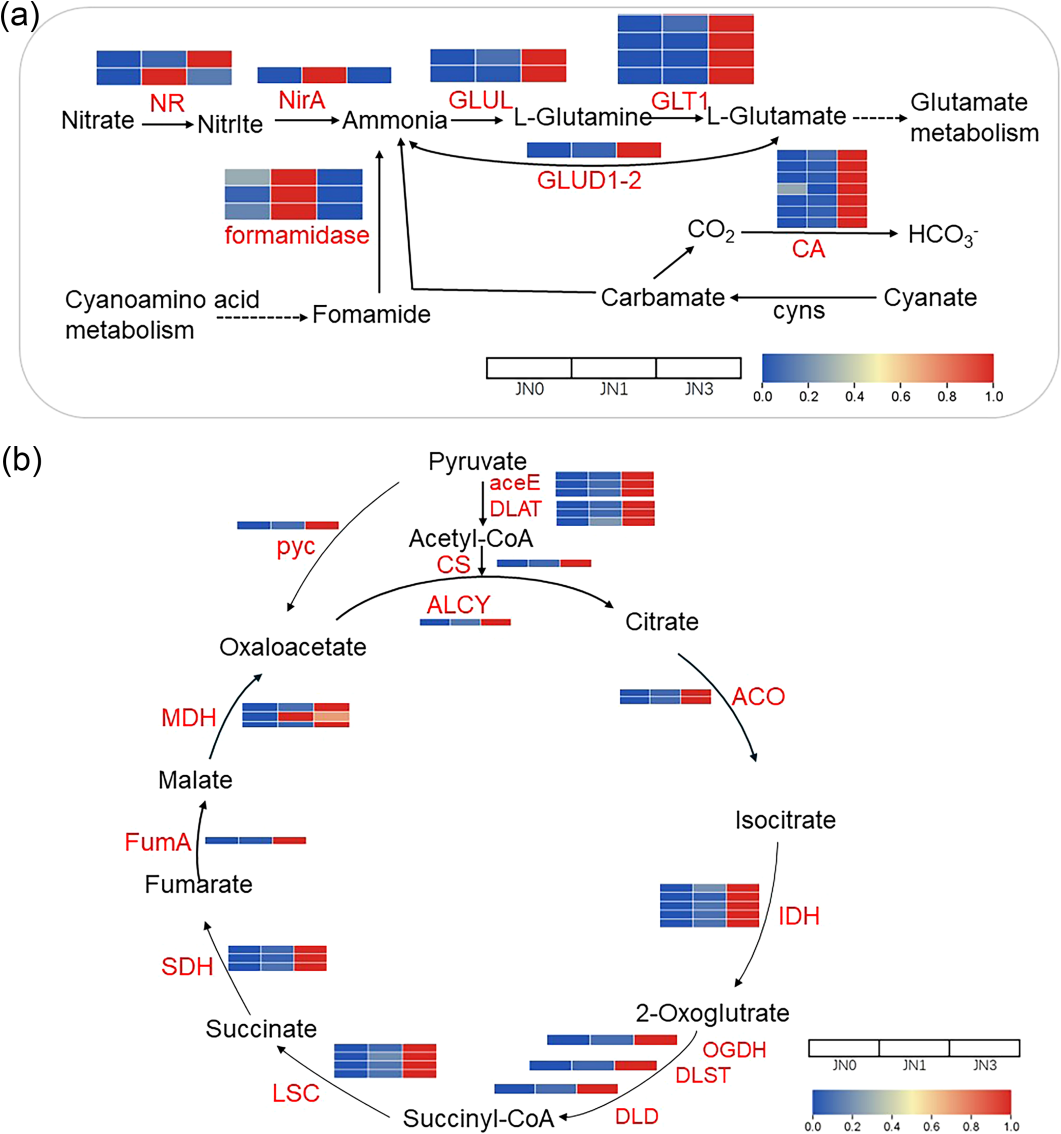
DEGs in *S. glauca* involved in **(a)** nitrogen metabolism and **(b)** the TCA cycle. JN0, JN1, and JN3 represent *S. glauca* under 0, 6, and 18 mg/kg peat addition, respectively.

Heatmap analysis further revealed that *VHA* family genes were consistently upregulated in both species under peat addition, indicating a conserved role in salt tolerance (**Figure 2f**). In contrast, *CHX* family genes displayed opposite expression trends between the two species: they were downregulated in *S. glauca* under the high peat but upregulated in *S. salsa* under both low and high peat concentrations. Notably, *SOS1*, a key gene mediating Na^+^ extrusion or xylem Na^+^ retrieval in salt-stressed plants, was specifically downregulated in *S. glauca* under the high peat condition, which may reflect a reduced demand for active Na^+^ efflux under ameliorated saline conditions. On the contrary, *NHX* and *HKT*, the key genes responsible for vacuolar Na^+^ sequestration and long-distance Na^+^ transport, were specifically upregulated in *S. salsa* (**Figure 3b**). The species-specific expression patterns of K^+^ channel genes (*AKT1* and *SKOR*) further highlighted divergent ion homeostasis strategies between the two species: under the high peat treatment, *S. glauca* exhibited upregulated expression of both *AKT1* and *SKOR*, which facilitated K^+^ homeostasis maintenance; in contrast, *S. salsa* showed upregulation of *AKT1* alone, with no differential expression of *SKOR* under peat treatments, suggesting a distinct mode of K^+^ regulation (**Figure 2f**).

### 3.5 DEGs in *S. glauca* involved in nitrogen metabolism and the TCA cycle

In *S. glauca*, the upregulated DEGs were mainly enriched in growth-related pathways, such as nitrogen metabolism and the TCA cycle. Based on KEGG enrichement analysis and Swiss-prot annotations, a total of 20 DEGs involved in nitrogen metabolism pathway were identified, including seven carbonic anhydrase (*CA*) genes, three formamidase genes, four glutamate synthase (*GLT1*) genes, two glutamine synthetase (*GLUL*) genes, two nitrate reductase (*NR*) genes, one glutamate dehydrogenase (*GLUD1-2*), and one ferredoxin-nitrite reductase (*NirA*) gene (**Figure 3a**). Expression analysis revealed that nine of these genes were upregulated under the low peat treatment, and 15 were upregulated under the high peat treatment. Only one gene, *Cluster-16558.28329_formamidase*, was downregulated under high peat addition but upregulated under the low peat condition. Moreover, four genes (*Cluster-16558.12787_GLT1*, *Cluster-16558.13182_GLUD1-2*, *Cluster-16558.13629_CA*, and *Cluster-16558.13839_GLUL*) were consistently upregulated under both low and high peat concentrations (**Figure 3a**; **Supplementary Table 3**).

A total of 30 DEGs involved in TCA cycle were identified, including five isocitrate dehydrogenase (*IDH*) genes, four succinyl-CoA synthetase alpha subunit (*LSC*) genes, three pyruvate dehydrogenase E1 component (aceE) genes, three dihydrolipoyllysine-residue acetyltransferase (*DLAT*) genes, three malate dehydrogenase (*MDH*) genes, three succinate dehydrogenase (ubiquinone) flavoprotein subunit (*SDH*) genes, two aconitate hydratase (*ACO*) genes, and one gene each for dihydrolipoyl dehydrogenase (*DLD*), citrate synthase (*CS*), ATP citrate (pro-S)-lyase (*ACLY*), 2-oxoglutarate dehydrogenase E2 component (dihydrolipoamide succinyltransferase) (*DLST*), 2-oxoglutarate dehydrogenase E1 component (*OGDH*), Fumarse (*FumA*) and pyruvate carboxylase (pyc) (**Figure 3b**). Of these, 16 genes were upregulated under the low peat treatment, and all 30 showed upregulation under the high peat condition. Notably, 16 genes, including three *MDH*, two *ACO*, two *aceE*, two *LSC*, and one each of *OGDH*, *SDH*, *DLD*, *DLST*, *DLAT*, *ACLY*, and *CS*, were upregulated under both low and high peat concentrations (**Figure 3b**; **Supplementary Table 4**).

### 3.6 DEGs of *S. salsa* involved in the photosynthesis and the biosynthesis of flavonoids, phenylpropanoids, and anthocyanins

**Figure 4:**
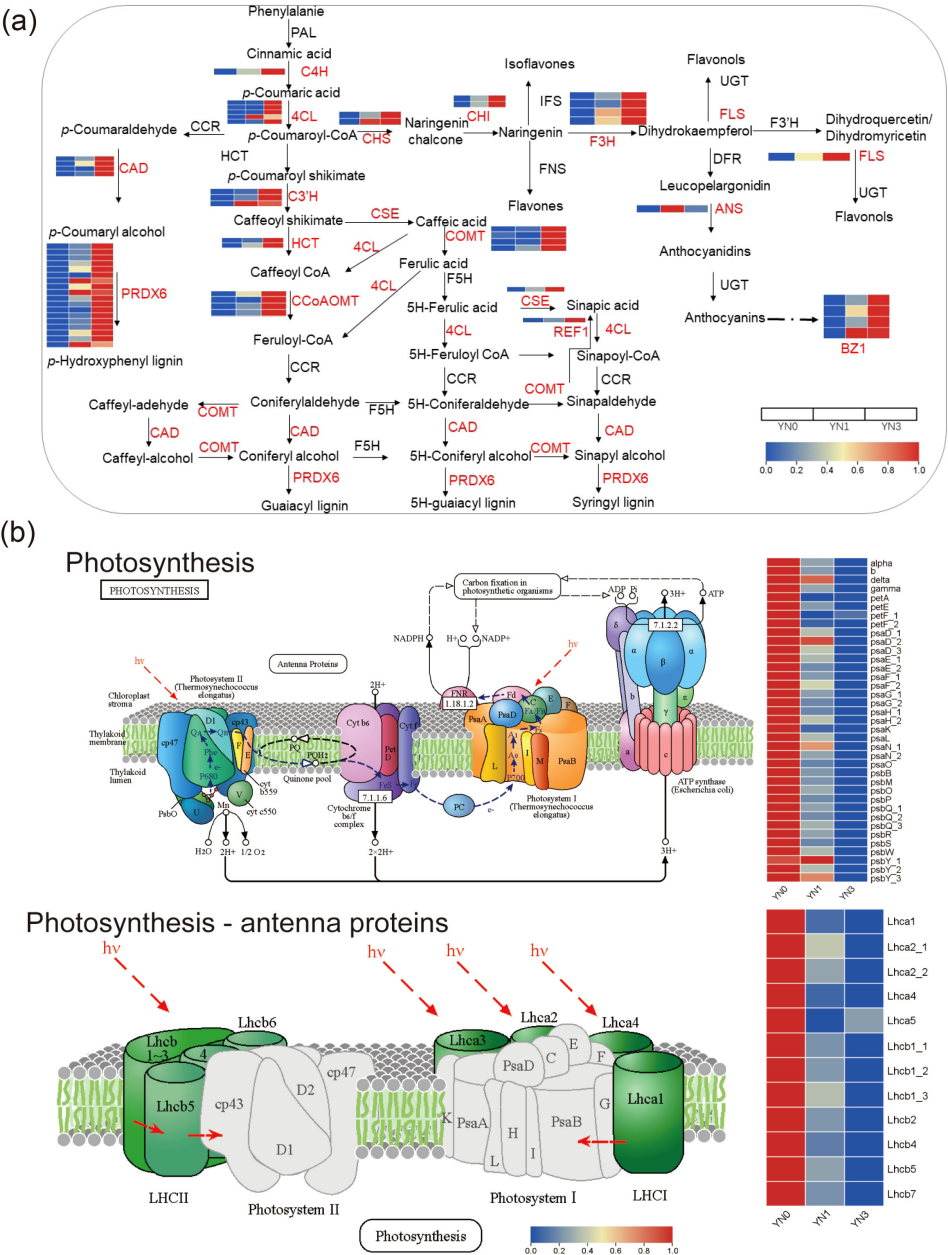
DEGs in *S. salsa* involved in **(a)** the biosynthesis of flavonoids, phenylpropanoids, and anthocyanins, and **(b)** photosynthesis. YN0, YN1, and YN3 represent *S. salsa* under 0, 6, and 18 mg/kg peat addition, respectively.

In *S. salsa*, the upregulated DEGs were mainly enriched in secondary metabolites-related pathways, including flavonoid, phenylpropanoid, and anthocyanin biosynthesis. According to KEGG enrichment analysis and Swiss-prot annotations, a total of 43, 20, and four DEGs were identified as being involved in phenylpropanoid, flavonoid, and anthocyanin biosynthesis, respectively (**Figure 4a**). Among the phenylpropanoid-related genes, six were upregulated under low peat addition, and 42 were upregulated under high peat addition. Five of which were upregulated across both low and high peat additions, including three *PRDX6* and two *C3’H* (**Figure 4a**; **Supplementary Table 5**). For flavonoid biosynthesis, eight and 19 DEGs were upregulated under low and high peat levels, respectively, with six genes upregulated under both conditions. These included two *CHS* and two *C3’H*, and one gene each for *F3H*, *FLS*, and *CHI* (**Figure 4a**; **Supplementary Table 6**). Regarding anthocyanin biosynthesis, four DEGs, all encoding *BZ1*, were upregulated specifically under high peat addition (**Figure 4a**; **Supplementary Table 7**).

In contrast, the downregulated DEGs in *S. salsa* were mainly enriched in growth-related pathways. Both low and high levels of peat addition caused the expression of genes related to photosynthesis, carbon fixation in photosynthetic organisms, etc. A total of 37 DEGs involved in the photosynthesis pathway were identified, including 16 DEGs related to photosystem I (*PsaD*, *PsaE*, *PsaF*, *PsaG*, *PsaH*, *PsaK*, *PsaL*, *PsaN*, and *PsaO*), 13 DEGs associated with photosystem II (*PsbB*, *PsbM*, *PsbQ*, *PsbP*, *PsbQ, PsbR, PsbS*, *PsbW*, and *PsbY*), four DEGs related to F-type ATPase (*b*, *delta*, and *gamma*), three DEGs involved in photosynthetic electron transport (*PetE* and *PetF*), and one DEG from the cytochrome b6/f complex (*PetA*). Additionally, 12 DEGs involved in the photosynthesis-antenna proteins pathway were identified, such as *Lhca1*, *Lhca2*, *Lhca4*, *Lhca5*, *Lhcb1*, *Lhcb2*, *Lhcb4*, *Lhcb5*, and *Lhcb7* (**Figure 4b**).

### 3.7 DEGs involved in the amino acid degradation pathway

Peat addition of peat suppressed the degradation and metabolism of amino acids in both *S. glauca* and *S. salsa*. Based on KEGG enrichment analysis and Swiss-prot annotations, 18 and 15 DEGs in *S. glauca* were involved in valine, leucine, and isoleucine degradation under low and high peat levels, respectively. Among them, 12 DEGs were downregulated at both peat levels, including three 3-methylcrotonyl-CoA carboxylase alpha subunit *(MCCC*), two aldehyde dehydrogenase (NAD+) (*ALDH*), two 2-oxoisovalerate dehydrogenase E2 component (dihydrolipoyl transacylase) (*DBT*), and one each of branched-chain amino acid aminotransferase (*ilvE*), isovaleryl-CoA dehydrogenase (*IVD*), 2-oxoisovalerate dehydrogenase E1 component subunit alpha (*BCKDHA*), and alanine-glyoxylate aminotransferase 2 (*AGXT2*) (**Supplementary Figure 4a**; **Supplementary Table 8**). In *S. salsa*, 10 and 18 DEGs were identified under low and high peat treatments, respectively. Seven of these were downregulated under both levels of peat addition, including three *MCCC*, and one each of *ALDH*, *DBT*, *IVD*, and *HPD1* (3-hydroxyisobutyrate/3-hydroxypropionate dehydrogenase) (**Supplementary Figure 4b**; **Supplementary Table 9**).

### 3.8 Expression profiling of transcription factors associated with salt tolerance

**Figure 5:**
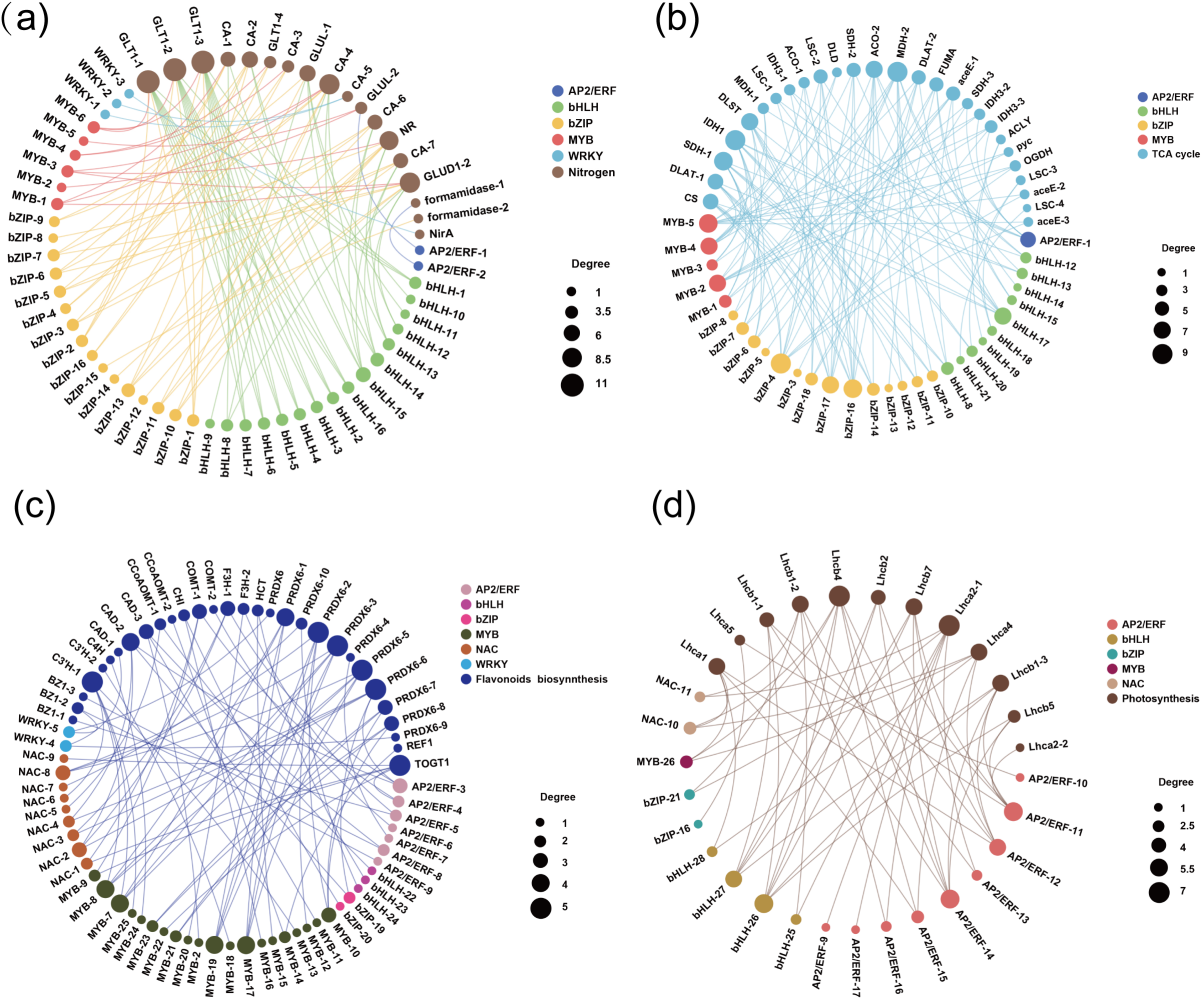
Correlation network analysis of transcription factors with genes involved in the **(a)** nitrogen metabolism of *S. glauca*, **(b)** the tricarboxylic acid (TCA) cycle of *S. glauca*, **(c)** the biosynthesis of flavonoids, phenylpropanoids, and anthocyanins of *S. salsa*, **(d)** the photosynthesis in *S. salsa*.

**Figure 6:**
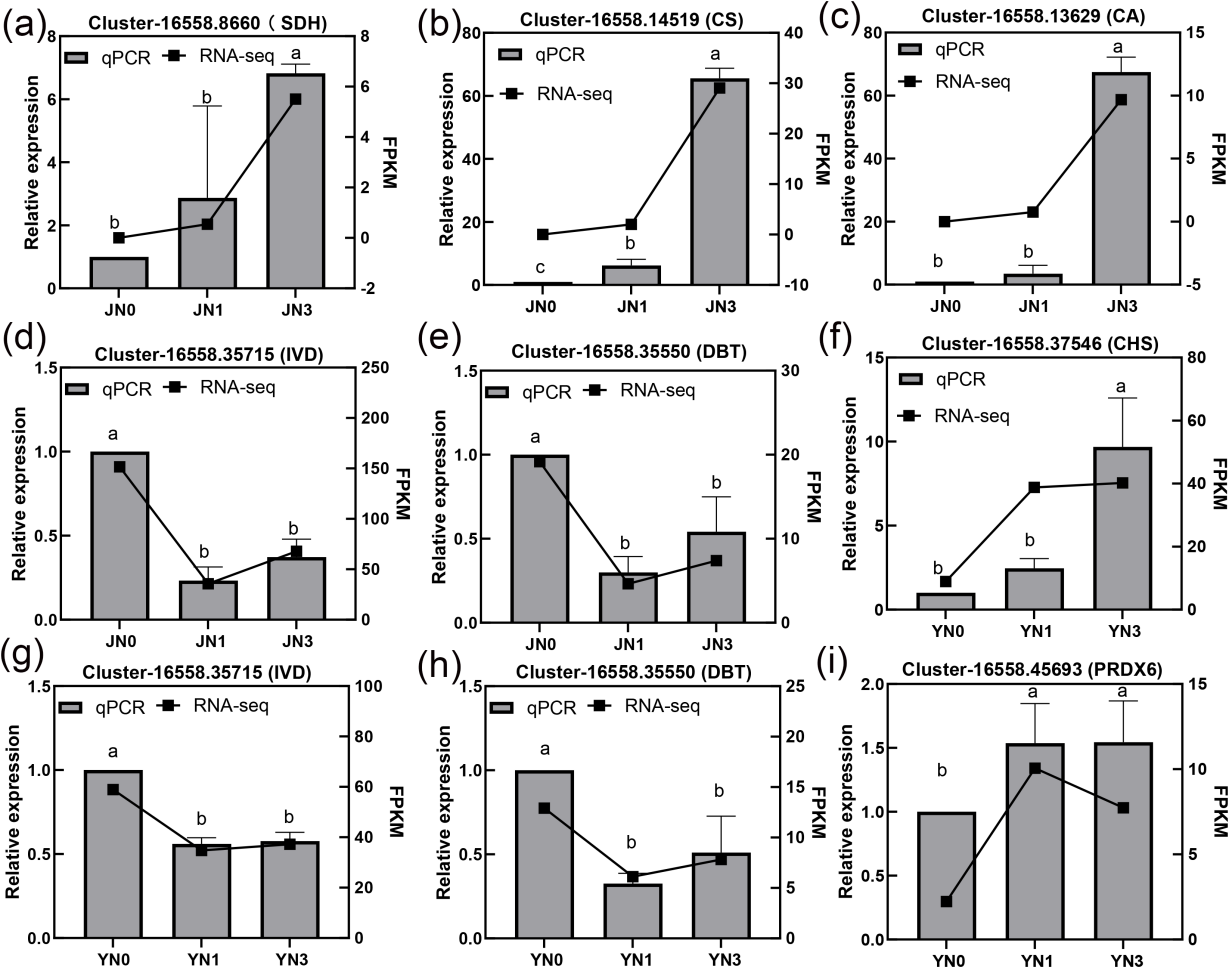
Validation of the expression of DEGs of core metabolic pathway genes via RT-qPCR. **(a–e)***S. glauca*; **(f–i)***S. salsa*. Different lowercase letters indicate significant differences among treatments (*p* < 0.05, Duncan’s multiple range test).

TFs such as WRKY, bHLH, bZIP, NAC, MYB, and AP2/ERF play crucial roles in regulating plant responses to abiotic stress. To better understand TFs involved in nitrogen metabolism, the TCA cycle, and the biosynthesis of flavonoids, phenylpropanoids, and anthocyanins, as well as amino acid degradation and photosynthesis, we conducted a correlation analysis between these TFs and relevant DEGs. This analysis identified 166 TFs, including 42 bHLH, 38 MYB, 30 bZIP, 29 AP2/ERF, 17 NAC, and 10 WRKY (**Supplementary Table 10**), which are closely associated with the key metabolic pathways mediating saline-alkaline tolerance and growth. In *S. glauca*, 25 TFs were detected under low peat addition and 82 under high peat addition, with 12 TFs shared between the two conditions (**Supplementary Figure 5**). Notably, 16 bHLH, 16 bZIP, 6 MYB, 3 WRKY, and 2 AP2/ERF TFs were significantly correlated with 18 nitrogen metabolism-related genes (e.g., *GLT1*, *GLUL*, *CA*), which are critical for nitrogen uptake and assimilation (**Figure 5a**; **Supplementary Table 11**). Additionally, 14 bHLH, 10 bZIP, five MYB, and one AP2/ERF TFs were tightly linked to 27 TCA cycle genes (e.g., *IDH, MDH, LSC*, **Figure 6b**; **Supplementary Table 12**), which provide energy precursors for growth. These TFs likely act as positive regulators of the growth-promoting pathway in *S. glauca*, directly driving the upregulation of nitrogen metabolism and TCA cycle genes under high peat concentration, thereby supporting biomass accumulation and root trait improvement.

In *S. salsa*, 19 TFs were identified under low peat addition and 86 under high peat addition, also with 12 shared TFs (**Supplementary Figure 5**). A total of 20 MYB, nine NAC, seven AP2/ERF, three bHLH, two WRKY, and two bZIP TFs were significantly correlated with 30 genes involved in the biosynthesis of flavonoids, phenylpropanoids, and anthocyanins (e.g., *PRDX6*, *C3’H, BZ1*)—the core stress-mitigating pathways in this species (**Figure 5c**; **Supplementary Table 13**). For the downregulated photosynthesis-related genes (e.g., *Lhca*, *Lhcb*), nine AP2/ERF, four bHLH, two NAC, two bZIP, and one MYB TFs were found to be correlated (**Figure 5d**; **Supplementary Table 14**), suggesting these TFs may mediate the transcriptional repression of photosynthetic pathways, allowing metabolic resources to be redirected toward secondary metabolite synthesis.

Notably, only one TF (*Cluster-16558.33486_MYB*) was present across all four libraries (**Supplementary Figure 5**), indicating that species-specific TF repertoires are responsible for the distinct transcriptional strategies of *S. glauca* and *S. salsa*. Furthermore, TFs involved in amino acid degradation suppression were identified in both species: in *S. glauca*, five AP2/ERF, two MYB, two bHLH, and one WRKY TFs were correlated with 20 genes involved in valine, leucine, and isoleucine degradation (e.g., *MCCC*, *ALDH, IVD*, **Supplementary Figure 4c**; **Supplementary Table 15**); in *S. salsa*, 13 AP2/ERF, nine MYB, eight bHLH, six NAC, six bZIP, and two WRKY TFs were linked to 20 amino acid degradation-related genes (e.g., *MCCC*, *ALDH*, *DBT*, **Supplementary Figure 4d**; **Supplementary Table 16**). This suggested that peat addition-induced suppression of amino acid degradation is also transcriptionally regulated by conserved and species-specific TF families, which helps conserve nitrogen resources and reduce energy consumption under salt stress. Collectively, these results revealed that high peat concentration modulates the expression of species-specific TFs, which in turn target the core metabolic pathways (growth-promoting pathways in *S. glauca* and stress-mitigating pathways in *S. salsa*) and the conserved amino acid degradation pathway.

### 3.9 RT-qPCR validation of core metabolic pathway genes

To validate the reliability of the RNA-seq data and clarify the expression patterns of key genes involved in core metabolic pathways, we performed RT-qPCR on 7 representative genes, including those associated with nitrogen metabolism (Cluster-16558.13629, *CA*), TCA cycle (Cluster-16558.8660, *SDH*; Cluster-16558.14519, *CS*), amino acid degradation (Cluster-16558.35715, *IVD*; Cluster-16558.35550, *DBT*), and secondary metabolite biosynthesis (Cluster-16558.37546, *CHS*; Cluster-16558.45693, *PRDX6*, **Figure 6**). The results showed that the relative expression levels of all detected genes by qPCR were completely consistent with their FPKM values from RNA-seq. Specifically, in *S. glauca*, growth-promoting pathway genes (*CA, SDH*, and *CS*) exhibited concentration-dependent upregulation (**Figures 6a–c**), their expression in high-peat (JN3) was significantly higher than in low-peat (JN1) and control (JN0), while amino acid degradation genes (*IVD*, and *DBT*) were downregulated (**Figures 6d, e**). In *S. salsa*, the expressions of secondary metabolite biosynthesis genes (*CHS*, and *PRDX6*) were notably upregulated, peaking in high peat (YN3). In contrast, the expressions of amino acid degradation genes (I*VD*, and *DBT*) were also downregulated (**Figures 6g, h**). Collectively, these results confirmed RNA-seq accuracy and validated the concentration-dependent regulation of key pathways and species-specific transcriptional strategies of the two *Suaeda* species under peat addition.

## 4 Discussion

Halophytes can thrive in saline-alkaline soil and are widely used in the restoration of such degraded environments (Flowers and Colmer, 2008; Zhao et al., 2024). In this study, high peat addition (18 g/kg) increased the aboveground, belowground, and total biomass of both *S. glauca* and *S. salsa.* Rich in organic matter, humic acid, and with a large specific surface area (Rezanezhad et al., 2016), peat improves soil organic matter content, reduces soil bulk density, and improves soil aggregation (Liu and Lennartz, 2018). Numerous studies have demonstrated that high concentrations of peat addition can enhance water retention capacity, salt leaching efficiency, improve cation exchange capacity, and activate nutrient availability via humic substances, and ultimately reducing salt accumulation in root-zone (Liu et al., 2021; Wang et al., 2014). These improvements in soil physicochemical properties may regulate soil water potential, thereby alleviating osmotic stress for *Suaeda* species. Importantly, our results further confirmed that peat addition directly reinforces intrinsic salt-tolerance mechanisms in halophytes (Li et al., 2023; Wang et al., 2019; Yang et al., 2021). Notably, peat concentration and species interacted significantly in regulating biomass (**Table 1**), reflecting species-dependent responses. High peat addition increased root traits and chlorophyll a/b contents exclusively in *S. glauca* (**Figures 1a, b, f, g**), consistent with the upregulation of porphyrin and chlorophyll metabolism and photosynthesis pathways (**Figure 2d**). In contrast, chlorophyll content in *S. salsa* remained stable, indicating its growth promotion primarily relies on stress-mitigation rather than photosynthetic enhancement.

Despite sharing basic tolerance mechanisms (leaf succulence, ion compartmentalization, osmotic adjustment, etc.), *S. glauca* and *S. salsa* exhibited inherent differences in their saline–alkali adaption strategies (Li et al., 2020; Wang et al., 2025a; Yang et al., 2008). Peat addition amplified these intrinsic strategies rather than inducing novel ones. At the ion regulation level, *S. salsa* would accumulate more shoot Na^+^ but less K^+^ than *S. glauca* (Wu et al., 2022), which aligns with our findings: *S. glauca* reduced Na^+^ uptake/transport via downregulating *CHX/SOS1* and enhanced K^+^ homeostasis via upregulating *AKT1/SKOR*, while *S. salsa* enhanced Na^+^ sequestration/transport via upregulating *NHX/HKT* and K^+^ absorption via upregulating *AKT1*. This ion regulation divergence was further reflected by the minimal shared DEGs (73 genes, 0.4%) between the two species, highlighting that peat addition exerts species-specific effects by reinforcing inherent adaptive frameworks.

During salinity adaptation, *S. glauca* primarily relies on nitrogen metabolism, whereas *S. salsa* relies more heavily on organic acid metabolism (Song et al., 2022). In the present study, high peat concentration induced species-specific transcriptomic responses that reinforced these pathways. *S. glauca* upregulated growth-related pathways, including nitrogen metabolism and the TCA cycle, while downregulating amino acid degradation and secondary metabolite-related pathways. In contrast, *S. salsa* upregulated secondary metabolite-related DEGs accompanied by the suppression of growth-related pathways.

Nitrogen, an essential element involved in synthesizing amino acids, proteins, and secondary metabolites, as well as regulating plant growth and stress signaling (Liu et al., 2024; Tian et al., 2022), plays a critical role in enhancing abiotic stress tolerance by regulating ion homeostasis, stabilizing cellular structures, and scavenging reactive oxygen species (ROS) (de Souza Miranda et al., 2015; Li et al., 2022; Singh et al., 2016; Tian et al., 2022; Wang and Lv, 2020). For *S. glauca*, nitrogen metabolism is a central metabolic pathway, with leaf amino acids accumulation (e.g., L-threonine and leucine) serving as osmolytes to alleviate osmotic stress (Song et al., 2022). Our results demonstrate that peat enhanced this pathway to promote *S. glauca* growth under saline-alkaline stress. High peat concentration upregulated 15 of 20 nitrogen metabolism-related DEGs (**Figure 3a**), including *GLT1*, *GLUL*, and *CA* genes critical for nitrogen uptake, assimilation, and osmoprotective amino acids. Concurrently, all 30 identified TCA cycle DEGs were activated (**Figure 3b**), including *IDH* and *MDH* that generate NADH/FADH2 for ATP production and provide carbon skeletons for nitrogen metabolism (Lorenz et al., 2007). This synergistic “carbon-nitrogen-energy” metabolic axis supported *S. glauca*’s biomass accumulation (**Figures 1c-e**) and root trait improvement (**Figures 1f, g**). Meanwhile, peat addition significantly suppressed 12 amino acid degradation-related DEGs across low and high concentrations, conserving nitrogen resources for protein synthesis and osmotic adjustment — a critical adaptation under salt stress (Slama et al., 2015). Collectively, high-concentration peat enhanced *S. glauca*’s salt tolerance by reinforcing nitrogen assimilation and energy production, while minimizing nitrogen loss via amino acid degradation.

In contrast, *S. salsa* improved tolerance by redirecting resources from growth to secondary metabolite biosynthesis, a strategy that effectively mitigates salt-induced oxidative stress. Flavonoids, phenylpropanoids, and anthocyanins are key ROS scavengers (Patil et al., 2024; Feng et al., 2023a). Among these, phenylpropanoids have been demonstrated to contribute to the higher salt tolerance of *S. salsa* than *S. glauca* (Yan et al., 2024). Flavonoids, as a major class of low-molecular-weight secondary metabolites, play a crucial role in protecting plants against various abiotic stresses such as drought, ultraviolet radiation, and salinity. Their primary function under stress conditions is the scavenging of ROS (Patil et al., 2024). In a recent study, flavonoids were reported to be involved in salt tolerance through ROS scavenging in the halophyte *Atriplex canescens*, and their content increased under saline conditions (Feng et al., 2023a). In *S. salsa*, analysis of widely targeted metabolites under saline conditions revealed an increase in flavonoid compounds such as quercetin, which act as antioxidants (Li et al., 2020; Li and Song, 2019).

High peat addition upregulated 42/43 phenylpropanoid-, 19/20 flavonoid-, and all 4 anthocyanin-related DEGs, including *CHS*, *C3’H*, and *BZ1* genes that synthesize ROS-scavenging compounds (Li et al., 2020; Patil et al., 2024). This observation is consistent with previous reports indicating that phenylpropanoids and flavonoids are central to the superior salt tolerance of *S. salsa* (Feng et al., 2023b; Li and Song, 2019; Yan et al., 2024). To support this stress-mitigating pathway, *S. salsa* downregulated 37 photosynthesis-related DEGs (e.g., *PsaD*, *Lhca*), reallocating energy from growth -related processes toward ROS scavenging. Despite this photosynthetic suppression, high peat still increased *S. salsa*’s biomass (**Figures 1c–e**), confirming that enhanced secondary metabolism effectively mitigates salt stress. RT-qPCR validation further confirmed the upregulation of key secondary metabolite genes (*CHS*, and *PRDX6*) and downregulation of amino acid degradation genes (*IVD*, and *DBT*) (**Figure 6**), reinforcing the reliability of this strategy.

A conserved response to high-concentration peat in both species was the suppression of amino acid degradation pathways, which likely contributes to salt tolerance by conserving nitrogen resources and reducing energy expenditure. Amino acids serve as key osmoprotective compounds in halophytes, as they reduce cellular osmotic potential to maintain water balance, protect cellular structures, and scavenge ROS (Slama et al., 2015). Previous research has shown that *S. salsa* accumulated more amino acids under high salinity stress (500 mM) than under low salinity (200 mM), which help the species to mitigate osmotic stress (Li and Song, 2019). Amino acid oxidation (e.g., valine, leucine, isoleucine) can supply energy under stress (Hildebrandt et al., 2015), but is energetically costly and depletes nitrogen reserves for osmoprotection (Slama et al., 2015). In this study, peat addition downregulated 12 amino acid degradation-related DEGs in *S. glauca* (e.g., *MCCC, ALDH*) and 7 in *S. salsa* (e.g., *MCCC*, *IVD)*. This suppression likely reflects improved soil nutrient availability from peat, which reduces plant dependence on amino acid catabolism for energy and allows the retention of amino acids for osmotic adjustment and protein synthesis. Furthermore, this pathway is transcriptionally regulated by conserved TF families (e.g., AP2/ERF, MYB) in both species (**Figure 5**). We infer that AP2/ERF represses degradation gene transcription by binding to ethylene-responsive elements (EREs) in their promoters, and that MYB reinforces this suppression through synergistic interaction, forming a conserved regulatory module induced by peat. This inference is consistent with the documented roles of these TF families in nitrogen metabolism (Liu et al., 2024).

Transcription factors play a central role in mediating the transcriptional responses of both species to high-concentration peat, forming a hierarchical regulatory network that targets core metabolic pathways. In *S. glauca*, 16 bHLH and 16 bZIP TFs were significantly correlated with nitrogen metabolism genes (e.g., *GLT1*, *GLUL*), and 14 bHLH and 10 bZIP TFs were linked to TCA cycle genes (e.g., *IDH*, *MDH*, **Figures 5a, b**). Based on previous studies (Liu et al., 2024), bHLH TFs may directly bind to E-box motifs in the promoters of *GLT1/GLUL* and *IDH/MDH* to activate transcription, whereas bZIP TFs may enhance this activation by forming heterodimers with bHLH proteins. Together, this synergistic TF module is proposed to drive the activation of growth-related pathways (Liu et al., 2024), aligning with the observed transcriptional upregulation and increased biomass in *S. glauca*. In *S. salsa*, 20 MYB and 9 NAC TFs were correlated with secondary metabolite biosynthesis genes (e.g., *CHS, C3’H*, **Figure 5c**), consistent with the well-documented role of MYB TFs in regulating flavonoid and phenylpropanoid pathways (Patil et al., 2024). Hence, we propose that MYB TFs specifically recognize ACGT-containing motifs in *CHS/C3’H* promoters to activate their expression, while NAC TFs may act upstream by inducing key MYB regulators. This hierarchical TF could amplify secondary metabolite biosynthesis to enhance ROS scavenging, consistent with the elevated flavonoid/anthocyanin accumulation inferred from our transcriptomic data. Additionally, 9 AP2/ERF TFs were correlated with downregulated photosynthesis genes (**Figure 5d**), suggesting they mediate the transcriptional repression of growth-related pathways to prioritize stress protection. Notably, only one TF (MYB family) was shared across all treatments (**Supplementary Figure 5**), emphasizing the species-specificity of TF regulatory networks. This TF-mediated regulation explains why peat enhances distinct metabolic pathways in the two species: it activates species-specific TF repertoires that amplify inherent adaptive strategies. Together, the TF-network and pathway analyses provide a comprehensive molecular framework for understanding how high-concentration peat improves saline-alkaline tolerance in *S. glauca* and *S. salsa*.

Overall, the practical significance of this study lies in: (1) identifying 18 g/kg as the optimal peat concentration for coastal saline-alkaline soils amelioration, providing quantitative parameters for practical remediation; (2) proposing a “species-specific phytoremediation strategy”: *S. glauca* is suitable for rapid vegetation establishment in early-stage salinization (enhancing soil organic matter through biomass accumulation), while *S. salsa* excels in stress mitigation in severely saline-alkaline soils (improving saline-alkaline tolerance via secondary metabolite biosynthesis). This strategy offers a theoretical basis for the precise selection of halophyte-amendment combinations. It can be applied to ecological remediation projects of saline-alkaline soils in southeastern coastal regions of China (e.g., Taizhou) and other areas with similar climatic conditions, contributing to the sustainable utilization of land resources.

## 5 Conclusion

**Figure 7:**
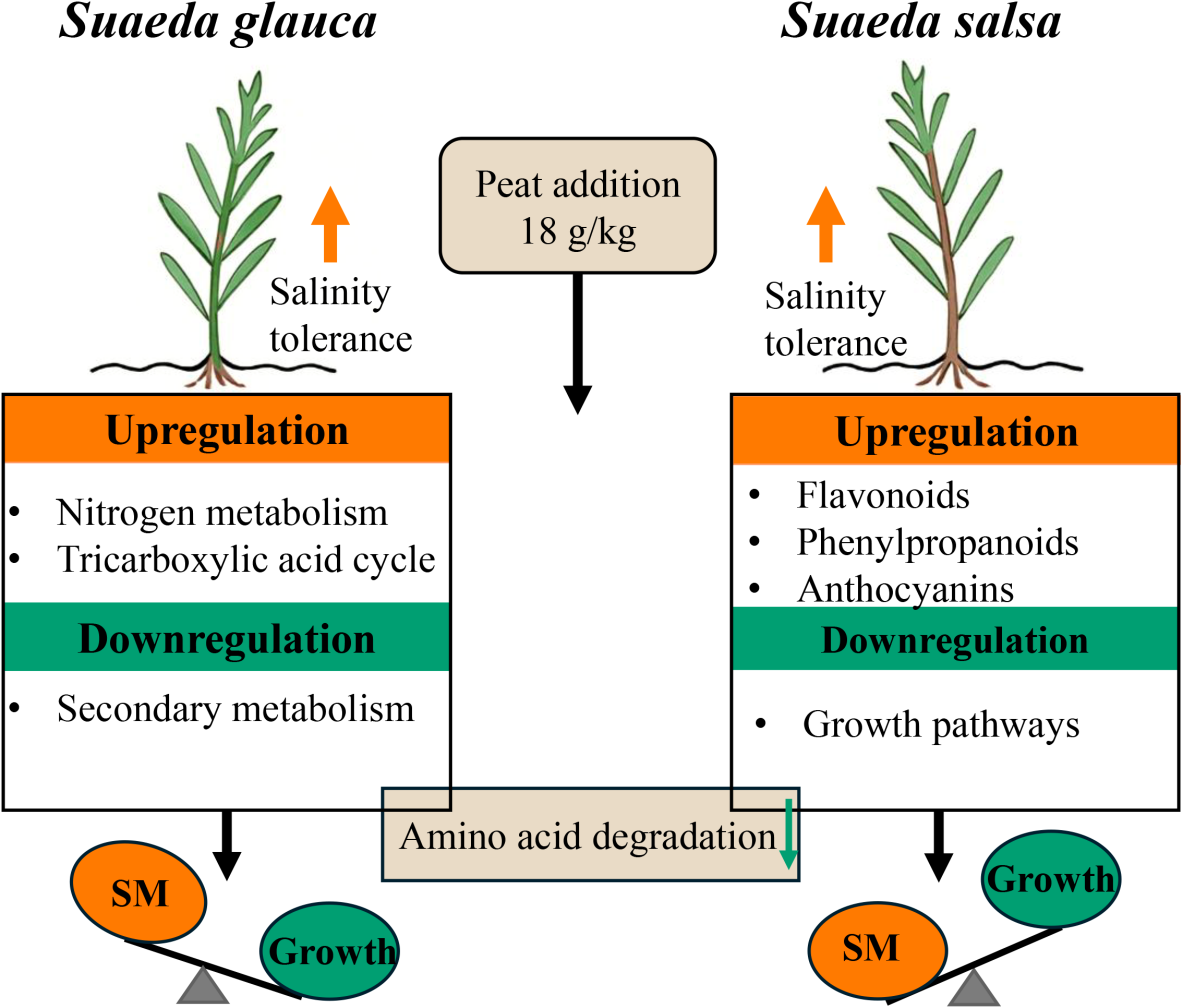
Model of high level of peat addition enhanced the salinity tolerance of *S. glauca* and *S. salsa.* SM indicated Secondary metabolism.

*S. glauca* and *S. salsa* exhibit distinct transcriptomic responses to peat addition during the remediation of saline-alkaline soils (**Figure 7**). Peat addition, particularly at a high concentration of 18 g/kg, significantly enhanced salt tolerance in both species. In *S. glauca*, the high peat concentration upregulated growth-related pathways such as nitrogen metabolism and the TCA cycle, while downregulating genes associated with amino acid degradation and metabolism, as well as secondary metabolite-related pathways. In contrast, *S. salsa* primarily upregulated genes involved in secondary metabolite-related pathways and downregulated growth-related pathways. Notably, the suppression of amino acid degradation–related genes under high peat addition was a conserved response in both species. These findings highlight species-specific adaptive strategies in halophytes, offering a foundation for identifying key salt-responsive genes and clarifying the distinct application potential of different halophytes in saline-alkaline soil remediation. Future studies should further validate the functions of these candidate genes under peat addition. In addition, as this study solely focused on leaf tissues, transcriptomic responses in roots warrant further investigations to fully elucidate whole-plant adaptive mechanisms during peat-assisted remediation of saline-alkaline soils.

## Data Availability

The datasets presented in this study can be found in online repositories. The names of the repository/repositories and accession number(s) can be found in the article/**Supplementary Material**.
