# Supplementary material for: High-concentration peat drives divergent transcriptomic responses to enhance saline-alkaline tolerance and phytoremediation in two Suaeda species
Source: Front Plant Sci. 2026 Feb 27;17:1761230. doi: 10.3389/fpls.2026.1761230 (PMC12984055; doi:10.3389/fpls.2026.1761230)
Supplement: Supplementary file 1 [file Image1.pdf]

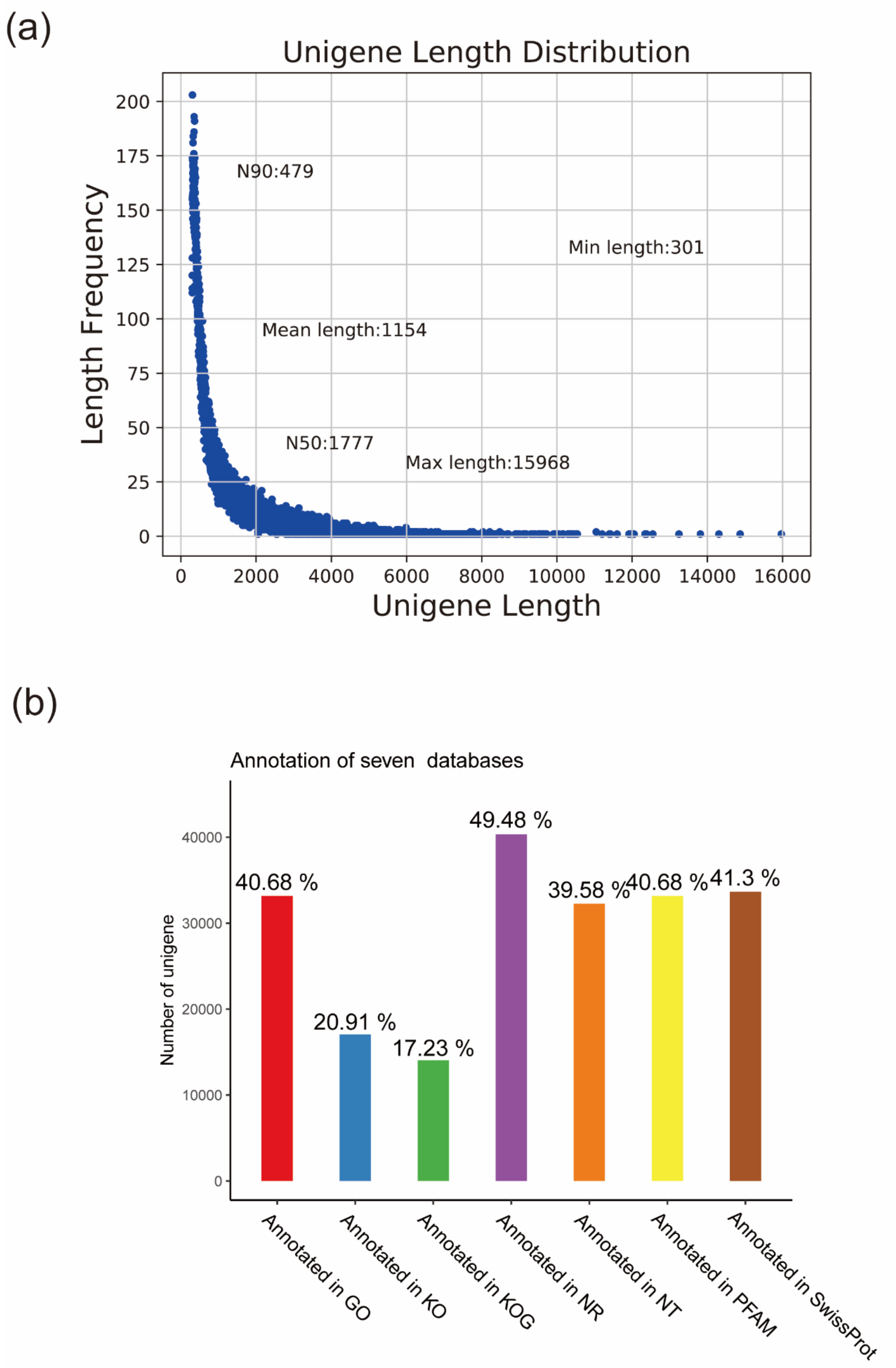

Fig. S1 Analysis of the transcriptome data. (a) length distribution of unigenes generated via de novo assembly; (b) unigenes annotated in seven databases.

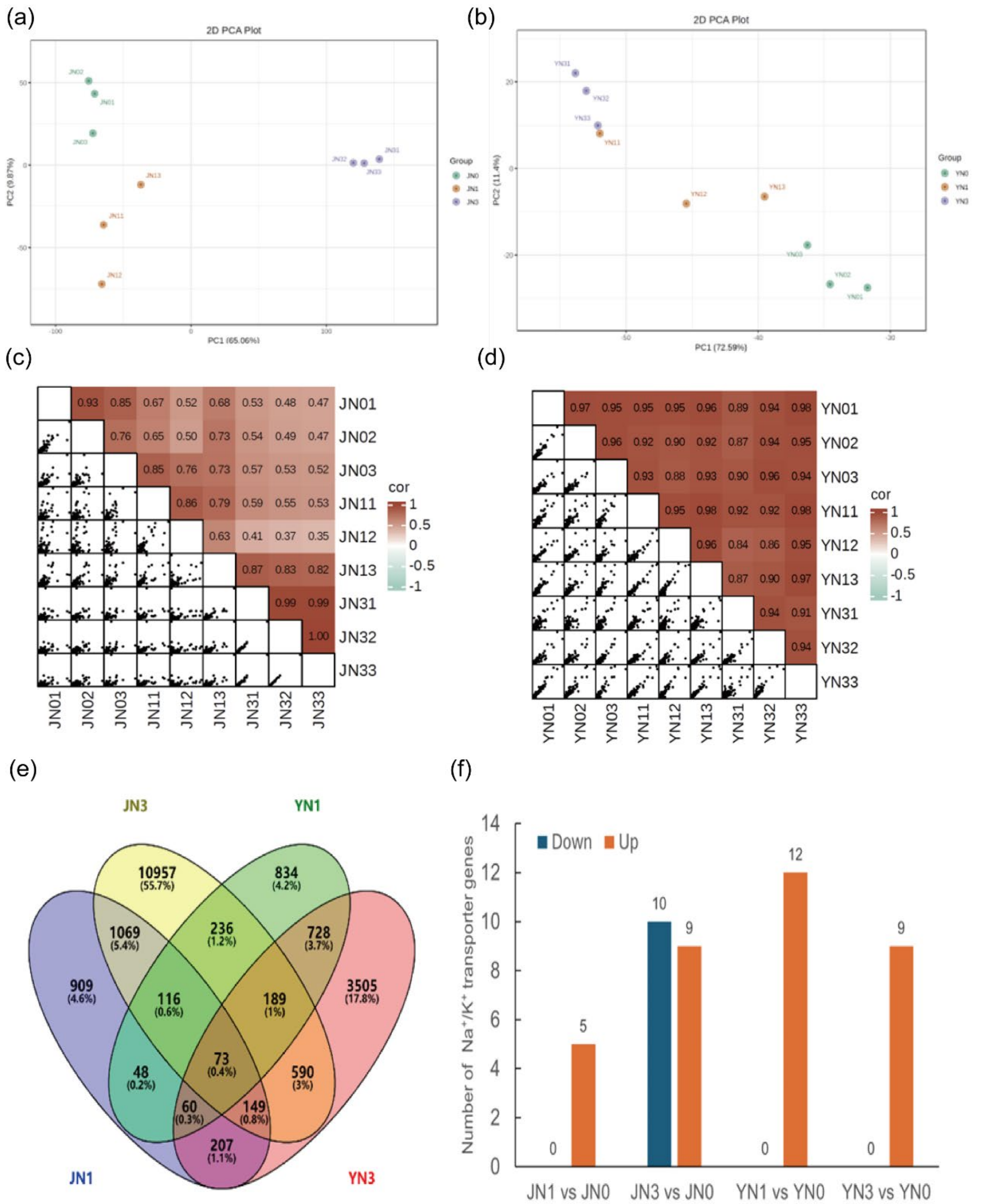

Fig. S2 (a and b) PCA plots for *S. glauca* and *S. salsa*, respectively; (c and d) Correlation analysis of biological replicates between different treatments for *S. glauca* and *S. salsa*, respectively; (e) Venny analysis of DEGs; (f) Number of Na+/K+ transporter genes. JN0, JN1, and JN3 represent *S. glauca* under 0, 6, and 18 mg/kg peat addition, respectively. YN0, YN1, and YN3 represent *S. salsa* under 0, 6, and 18 mg/kg peat addition, respectively.

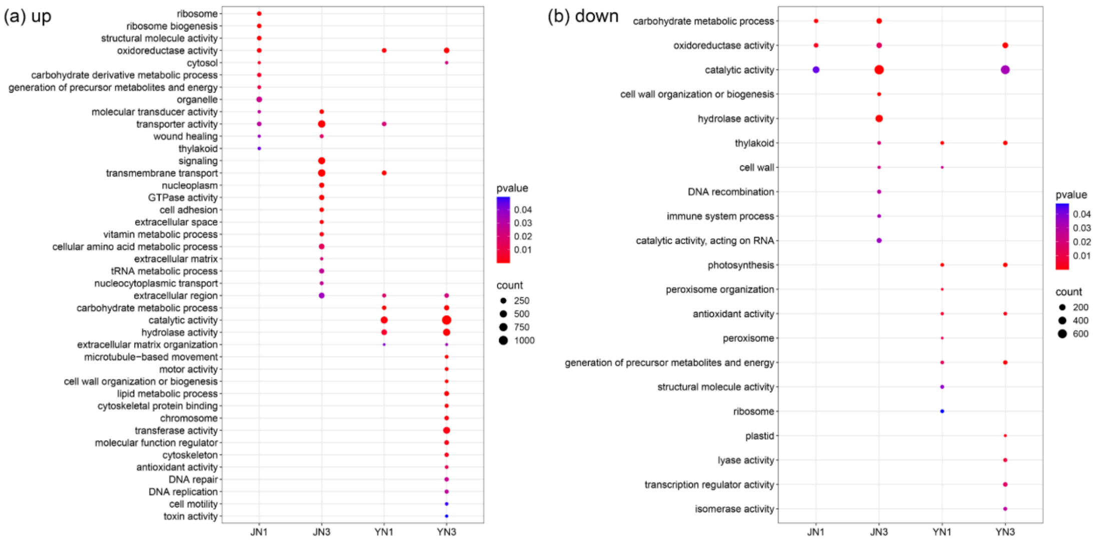

Fig. S3 GO enrichment results showed that the responses plants to peat addition differed among *S. glauca* (JN) and *S. salsa* (YN). (a) up-regulated DEGs; (b) down-regulated DEGs.

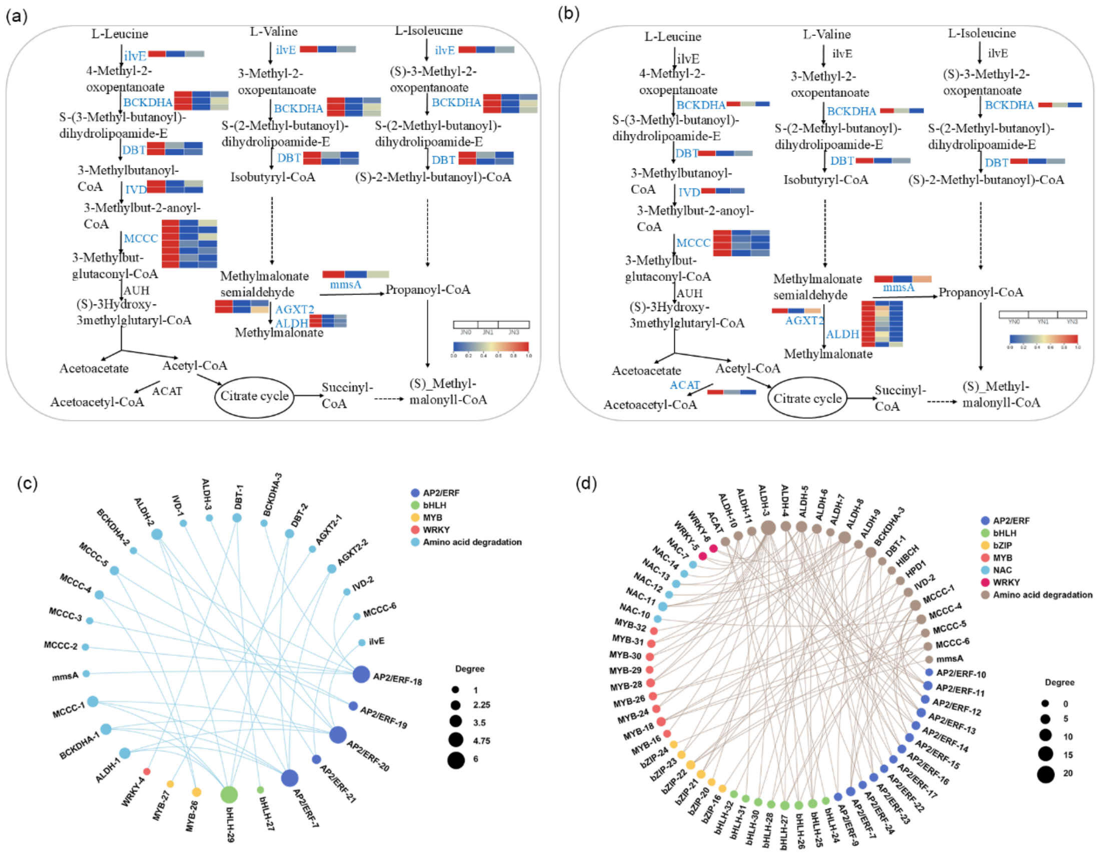

Fig. S4 Amino acid degradation pathway in (a) *S. glauca* and (b) *S. salsa.* Correlation network analysis of transcription factors with genes involved in the amino acid degradation in (c) *S. glauca* and (d) *S. salsa,* respectively. JN0, JN1, and JN3 represent *S. glauca* under 0, 6, and 18 mg/kg peat addition, respectively. YN0, YN1, and YN3 represent *S. salsa* under 0, 6, and 18 mg/kg peat addition, respectively.

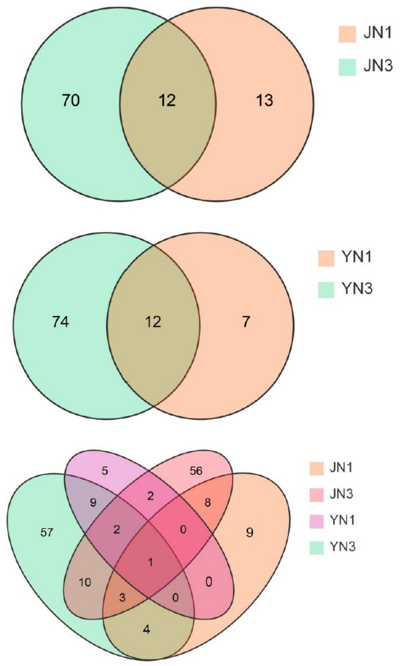

Fig. S5 Venny program of transcription factors identified in *S. glauca* (JN) and *S. salsa* (YN).
